# The role of physical activity on healthcare utilization in China

**DOI:** 10.1186/s12889-023-16625-4

**Published:** 2023-11-30

**Authors:** Xiao-Lin Lei, Ke Gao, Huan Wang, Wei Chen, Gen-Rui Chen, Xing Wen

**Affiliations:** 1 Department of Cardiology, Xijing Hospital, Air Force Military Medical University (Fourth Military Medical University), Xi’an, 710032 China; 2 Department of Cardiology, The First Affiliated Hospital of Xi’an Jiaotong University, Xi’an, 710061 China; 3 The Center of Gastrointestinal and Minimally Invasive Surgery, Department of General Surgery, The Third People’s Hospital of Chengdu &The Affiliated Hospital of Southwest, Jiaotong University, Chengdu, 610032 China

**Keywords:** Physical activity, Healthcare utilization, Healthcare expenditure, Catastrophic health expenditures, Public health

## Abstract

**Background:**

Evidence on the role of physical activity (PA) on healthcare utilization and expenditure is limited in China. We aimed to examine the association between the total physical activity (TPA) per week, healthcare service use and expenditure.

**Methods:**

We extracted the data from China Health and Retirement Longitudinal Study (CHARLS) 2011, 2013, and 2015. Participants more than 50 years old who completed the follow-up for the three waves were enrolled. We converted the volume of vigorous physical activity (VPA) into an equivalent volume of moderate physical activity (MPA) and calculated the TPA per week for each participant. 12,927 of the 17,708 participants in CHARLS were included in our analysis. More than one-third of participants over 50 years old never participate in any moderate or intensity activity, and the median of self-reported moderate or intensity PA was about 525 (IQR 0–1680) MET-minutes per week in 2015.

**Results:**

Compared to inactive subjects, the highest level of TPA was significantly related to the decreased risk number of inpatient visits (IRR: 0.58; 95% CI:0.50–0.67, *p* < 0.001), inpatient hospital days (IRR: 0.60; 95% CI: 0.42–0.84, *p* < 0.01), healthcare expenditure (IRR: 0.71; 95% CI: 0.65–0.79, *p* < 0.001) and catastrophic health expenditures (OR: 0.57; 95% CI: 0.45–0.72, *p* < 0.001) after adjusting for covariates.

**Conclusions:**

Engaging in moderate-to-vigorous PA may drive a potential decrease in healthcare utilization, healthcare expenditure and household financial risk with a dose–response relationship in China, and some possible policy implications in public health may be considered to promote exercise in the middle-aged and elderly to reduce the medical burden on individuals and healthcare systems.

**Supplementary Information:**

The online version contains supplementary material available at 10.1186/s12889-023-16625-4.

## Introduction

Physical activity (PA) confers multiple health benefits in the prevention and management ofelicits multiple health benefits in preventing and managing many chronic non-communicable diseases, encompassing cardiovascular diseases, depression, metabolic syndrome, and cancer, and it is also closely related to life expectancy and all-cause mortality [1–3]. On the account of a systematic review of scientific reports, the 2018 PA guidelines for Americans recommend that adults are supposed to do 150–300 MET-minutes of moderate-intensity aerobic exercise at least, or 75–150 MET-minutes of high-intensity aerobic exercise, or their incorporation each week [4]. Despite the health benefits, about 80% of adults and juveniles are lack of physical activities, however, sufficient PA is reported by merely 6% of men, 19% of women, and 20% of adolescents [4]. People in economically advanced regions in China currently engage in more physical activities than those in less economically. However, the data from the China Health and Nutrition Survey (CHNS) demonstrated that the amount of PA in China was decreased significantly not only in occupational domains but also in domestic domains from 1991 to 2011 [5].

The most common causes of NCDs are behavioral risk factors and metabolic, which can be largely preventable by the risk factors of self–management, including tobacco and alcohol consumption, physical activity, weight, food, and dental health care. The preventable of NCDs is focus on the role of individual responsibility to manage the risk factors of NCDs [6]. The entire cost to the healthcare system of physical inactivity is estimated at $53.8 billion worldwide in 2013 [6]. Among all countries, chronic non-communicable diseases are the main contributor to healthy and economic burden [7, 8]. In China, according to a recent research, physical inactivity contributes to 12%-19% of the risk of five chronic diseases, namely stroke, hypertension, cancer, diabetes and coronary heart disease. Moreover, in China, inadequate physical activity accounts for about 15 percent of the medical and non-medical costs of these chronic diseases each year [9]. Lee et al. showed that about 1% of the burden of disease from coronary heart disease, 6.4% of type 2 diabetes, 8.4% of breast cancer, and 8.3% of all-cause mortality in Chinese population were on the account of physical inactivity in 2011 [10]. Conservatively projected, physical inactivity led to a $4.8 billion cost of Chinese healthcare system in 2013 [6]. Despite the enormous economic burden of physical inactivity, the effect of PA levels on long-term healthcare utilization was given little attention in China.

In this population-based panel data study, we extracted the sample data from China Health and Retirement Longitudinal Study (CHARLS) 2011, 2013, and 2015, and restricted our sample to respondents aged 50 or older, with the sample size of 12,927 of the 17,708 participants. By performing further regression analyzes using logistic regression models and/or negative binomial regression models, we compared the association of time per week with TPA and number of hospital visits, hospital days, total medical costs, and catastrophic medical expendituresTPA. Moreover, random-effects logistic regression and negative binomial regression were adopted to estimate the relationship between different levels of TPA and healthcare utilization. Furthermore,, we examined the relationship between the level of household healthcare expenditures and TPA levels, including the total healthcare expenditure and the risk of catastrophic health expenditure (CHE) of households.

## Materials and methods

### Study design and data source

Data for this study were taken from the China Health and Retirement Longitudinal Study (CHARLS) [11], which encompasses rich information about healthcare utilization, healthcare expenditures, health insurance status, and sociodemographic characteristics. CHARLS is a Multistage Stratified Probabilistic Proportional Sampling cohort, and interviews were repeated every two years. We used information from the first three waves of 2011, 2013 and 2015. We restricted our sample to respondents aged 50 or older, with the sample size of 12,927 of the 17,708 participants in CHARLS. The Biomedical Ethics Review Committee of Peking University approved CHARLS, and all participants were required to provide written informed consent. The CHARLS aims to collect a high quality nationally representative sample of Chinese residents ages 45 and older to serve the needs of scientific research on the elderly. The baseline national wave of CHARLS is being fielded in 2011and includes about 10,000 households and 17,500 individuals in 150 counties/districts and 450 villages/resident committees. The individuals will be followed up every two years. All data will be made public one year after the end of data collection.

### TPA

As described in the codebook, PA is classified into 3 levels based on intensity: vigorous physical activity (VPA) (i.e., PA that makes you feel short of breath, such as lifting heavy objects, farming, aerobic exercise, fast cycling, eetc.), moderate physical activity (MPA) (i.e., PA that allows you to breathe faster than usual, such as carrying light objects, walking fast, cycling at a normal speed, etc.), and low-intensity physical activity (LPA) (i.e., walking from one place to another at home or work, as well as other recreational, athletic, and leisurely perambulations). Participants were asked to report exercise intensity, frequency, and duration per week. Frequency: The responses ranged from 0–7 days per week, and PA frequency was categorized as approximately 0 (never), 1 to 2, 3 to 5, 6 to 7 days every week. Duration: the length of each PA was stratified into 5 levels, including PA less than 30 min, 30 to 119 min, 120 to 239 min and longer than 240 min each session. Volume: The total length of each PA intensity was assessed by: i) estimating the daily average duration for each physical activity; ii) Volume of PA calculated by multiplying frequency by the duration of each PA intensity. Instead of using minutes per week, we adopted metabolic equivalent value (MET) minutes per week for better applicability of our findings. According to previous studies [4], 1 MET refers to oxygen consumption at rest, VPA can be expressed as 8 METs, MPA can be expressed as 4 METs, and LPA can be expressed as 3.3 METs. TPA is calculated as the sum of VPA + MPA + LPA. The main study exposure, TPA, has been categorized into four levels: 0, 15–599, 600–1999 ≥ 2000 MET-minutes/week, according to the tertiles of TPA.

### Healthcare utilization and expenditure

Participants were asked to report their use of healthcare services in CHARLS. Self-reported information by participants was collected on how much respondents paid in total and how much they paid out of pocket (deducting the reimbursed expenses) for their inpatient visits during the past year and for outpatient visits during the past month. We calculate the annual out-of-pocket paid for outpatient care as the monthly spending times 12 for each participant.

Likewise, CHARLS records the same information for spouses of all participants; therefore, the out-of-pocket paid data for spouses was used to calculate catastrophic health expenditures (CHE) at the household level. When out of pocket payment on health care was more than or equal to 40% of a household’s payment capacity, a household was defined as incurring CHE [12]. A household’s payment capacity was defined by deducting the food-based expenditure of the household from the total consumption household spending.

### Covariates

The study covariates included participants' demographic variables and lifestyle behaviors. Socio-demographic characteristics were composed of age, sex, marital status (unmarried, married and partnered, and others), education (college and above, secondary school, and primary school and below), residence (urban, rural), and economic status (total household annual expenditure per capita) quartiles. Lifestyle behaviors included body mass index (BMI, categorized into obese BMI ≥ 28 kg/m^2^, overweight 24 ≤ BMI < 28 kg/m^2^, normal weight 18.5 ≤ BMI < 24 kg/m^2^, and underweight BMI < 18.5 kg/m^2^), smoking status (former and current smokers, never), and drinking status (former drinker, lifetime abstainer, current light to moderate drinker and current heavy drinker). Annual per-capita household consumption spending was used as a proxy for socioeconomic status. We stratified socioeconomic status into 4 levels according to quartiles of per-capita household consumption expenditure last year (quartile 1, 0–4999 CNY; quartile 2, 5000–9999 CNY; quartile 3, 10,000 CNY or more). The number of non-communicable diseases (NCDs), composed of diagnosed hypertension and eleven self-reported diagnosed chronic diseases (dyslipidemia, diabetes, chronic lung disease, heart disease, stroke, liver disease, digestive disease, kidney disease, arthritis, asthma and cancer) for each participant was included in the assay.

### Statistical analysis

Firstly, logistic regression with cross-sectional data was used to examine the associations between TPA and the presence of catastrophic health expenditures in 2015; negative binomial regression models were performed to study the relationship between different levels of TPA and the number of inpatient visits times, inpatient hospital days, outpatient visit times, and healthcare expenditure in 2015. Instead of the Poisson regression model, the Negative binomial model was chosen to allow for overdispersion as the variances greater than the means of outcome variables [13].

Second, random-effects logistic regression and negative binomial regression were adopted to estimate the relationship between different levels of TPA and healthcare utilization. Since CHARLS introduced new respondents to guarantee the sample representativeness for some respondents may be dead or lost. To rule out this problem, we use unbalanced panel data. For regression analysis with cross-sectional data and panel data, we report associations as incidence rate ratios (IRR) and odds ratios (OR) adjusted for age, sex, BMI, the number of non-communicable diseases, education, marital status, socioeconomic status, residence, smoking status, drinking status.

We employed a restricted cubic spline (RCS) logistic regression with five knots (5th, 27.5th, 50th, 72.5th and 95th quantiles) to investigate the non-linear relationships between physical activity and health service. Additionally, we adjusted for potential confounding factors including age, sex, BMI, the number of non-communicable diseases, education level, marital status, socioeconomic status, residence type, smoking status and drinking status in the RCS logistic regression analysis.

Stata version 16.0 and R version 4.0.2 were used for conducting statistical analyses, and a two-sided *P* value of < 0.05 was considered statistically significant.

## Results

### Sample characteristics

We analyzed 12,927 of 17,708 participants in CHARLS and three waves of follow-up information. The participants’ demographic characteristics, health care utilization and cost were presented in the appendix (S1 Table). The median age of participants was 62 years (IQR 56–69). 6086 (48%) participants were male, 6496 (52%) were female. More than one-third of participants over 50 years old never participate in any moderate or intensity activity, and the median of reported moderate or intensity PA was about 525 (IQR 0–1680) MET-minutes per week in 2015.

**Fig. 1:**
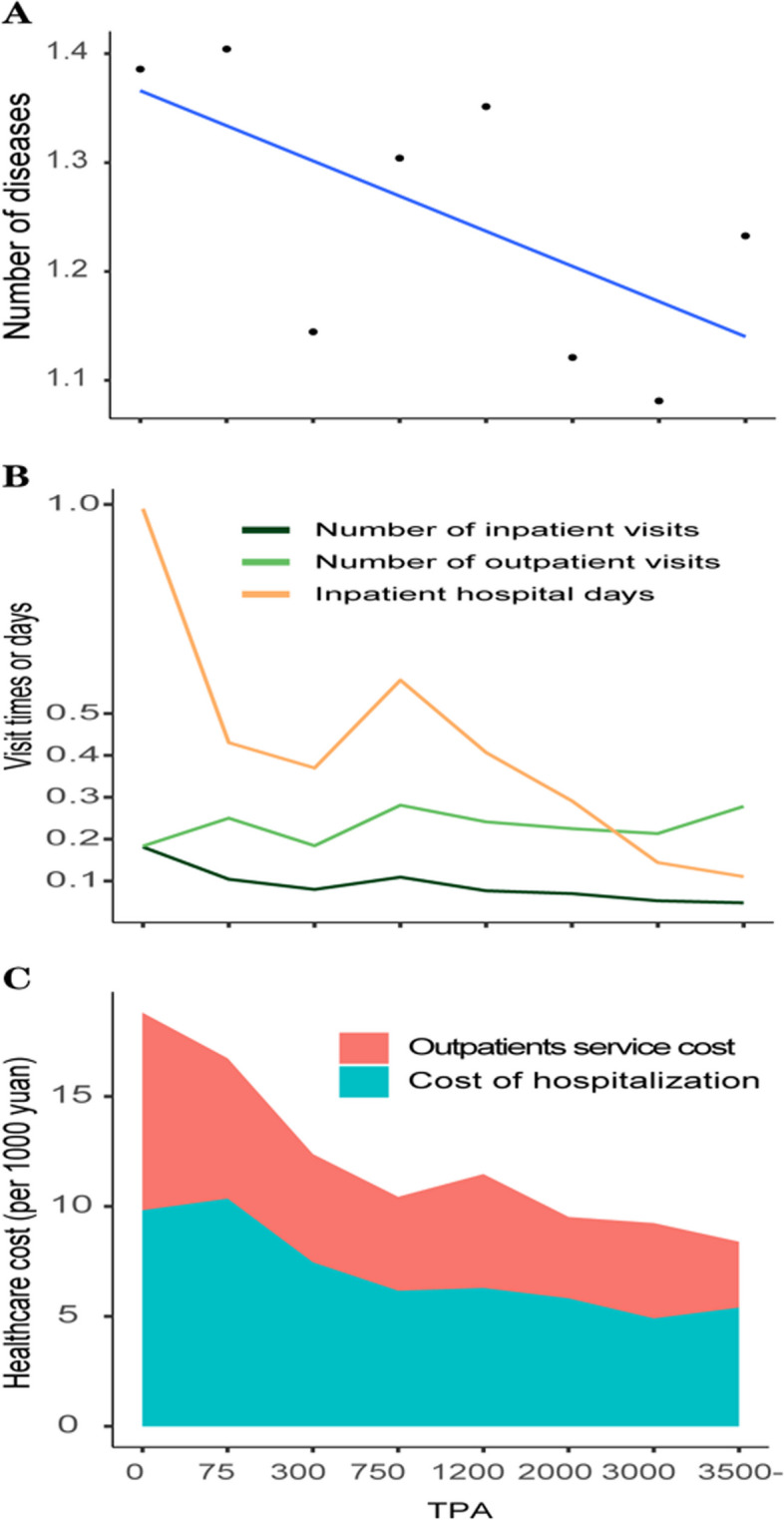
The association between physical activity volume and health condition and health care cost. **A** the mean number of diseases at different levels of TPA. **B** the health care utilization at different levels of TPA. C, the mean cost of health care at different levels of TPA in China. TPA = total physical activity per week

The number of diseases presented an overall trend of mild decrease with the time of TPA per week increasing within a certain range in 2015 (Fig. 1A). As the time of TPA per week increases, the number of inpatient visits and inpatient hospital days has decreased, and also the case for healthcare expenditures, including both outpatient and inpatient expenditures (Fig. 1B-C).

### Impact of TPA on healthcare utilization

**Fig. 2:**
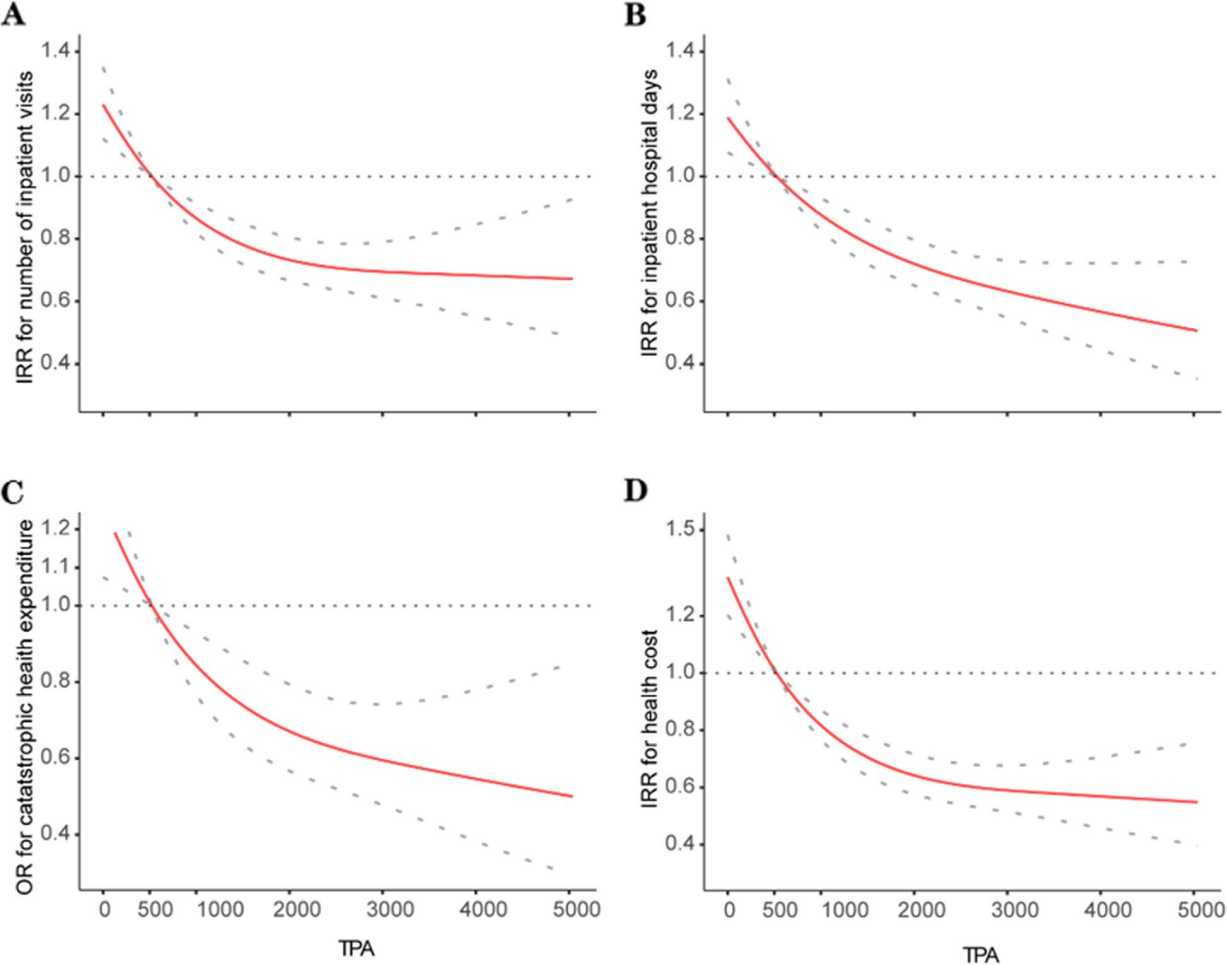
The association between physical activity and health service use and cost in China, 2015. Restricted cubic splines adjusted covariates (age, sex, BMI, the number of non-communicable diseases, education, marital status, socioeconomic status, residence, smoking status, drinking status) showed a curvilinear relationship between TPA (as a continuous variable) and risk of the number of inpatient visits **A** inpatient hospital days **B** catastrophic health expenditures **C** and health care cost **D** Red lines represent the hazard ratio, gray lines represent the 95% confidence intervals. TPA = total physical activity per week. BMI = body mass index

By performing further regression analysis using the logistics regression model and/or negative binomial regression models, we found that the time of TPA per week was significantly associated with the number of inpatient visits, inpatient hospital days, total healthcare cost and catastrophic health expenditures (Fig. 2, S1 and S2 Figs), except for the number of outpatient visits (S3 Fig). And the restricted cubic splines to flexibly model adjusted covariates described in the Methods showed that there was a curvilinear relationship between the time of TPA per week (as a continuous variable) and risk of inpatient visits (P for nonlinearity < 0.001, Fig. 2A), inpatient hospital days (P for nonlinearity < 0.001, Fig. 2B), catastrophic health expenditures (P for nonlinearity < 0.001, Fig. 2C) and total health care cost (P for nonlinearity < 0.001, Fig. 2D).

**Fig. 3:**
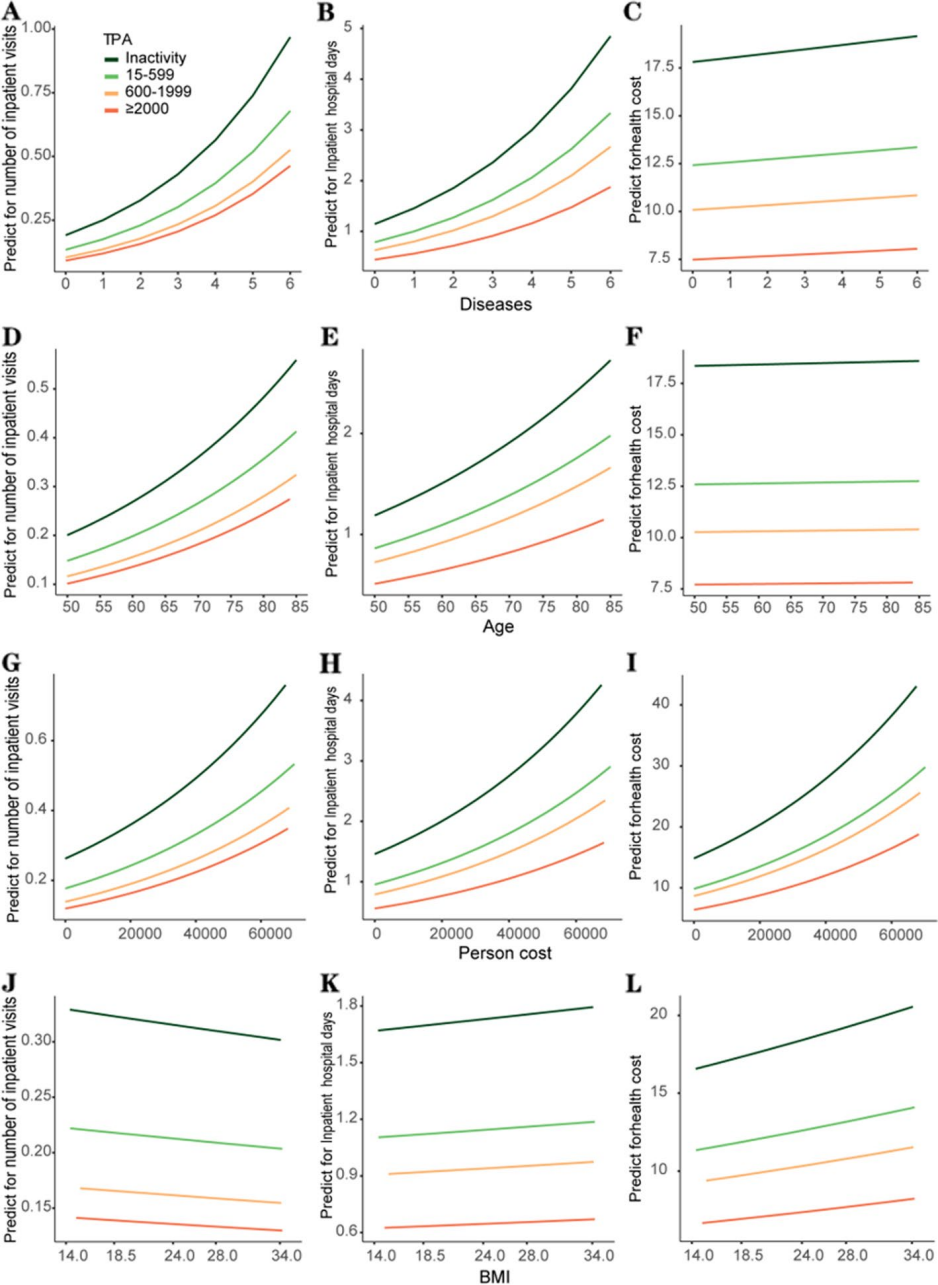
Relation between health indicators and predicted values for health care utilization and cost in China 2015. The number of inpatient visits, inpatient hospital days and total healthcare cost have risen as age increased **A**-**C** Such trends in health care utilization and cost could also be observed in personal spending and non-communicable diseases **D**-**I** Inpatient hospital days and total health care cost, but not the number of inpatient visits, have risen linearly as BMI increased **J**-**L** TPA has been categorized into four levels: 0, 15–599, 600–1999, ≥ 2000 MET-minutes/week. TPA = total physical activity per week

As expected, the number of inpatient visits, inpatient hospital days, and total health care costs were increasing with increased number of non-communicable diseases age (Fig. 3A-C). Such trends in healthcare utilization and expenditure could also be observed in Age and personal spending (Fig. 3D-I). Inpatient hospital days and total health care cost, but not the number of inpatient visits, rose linearly as BMI increased (Fig. 3J-L). More interestingly, all curves signal an important phenomenon: the higher level of TPA, the lower health care utilization and cost for participants of any age, number of NCDs, BMI and personal social-economic status.

**Fig. 4:**
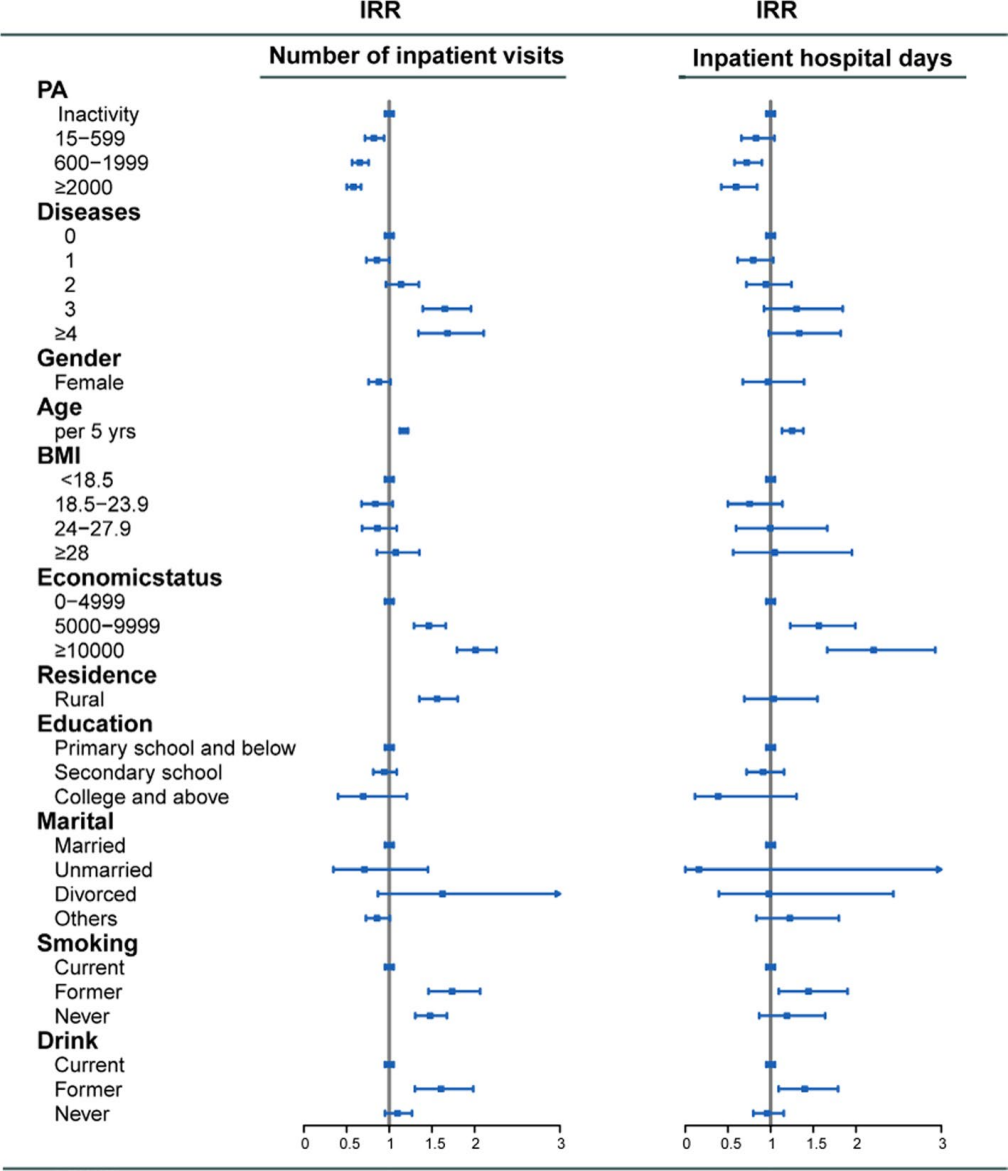
The association between physical activity and health service use, in China, 2011–2015. IRR was adjusted for age, sex, BMI, the number of non-communicable diseases, education, marital status, socioeconomic status, residence, smoking status, drinking status. Total time of moderate-intensity physical activity per week has been categorized into four levels: 0, 15–599, 600–1999, ≥ 2000 MET-minutes/week. IRR = incidence rate ratio

**Fig. 5:**
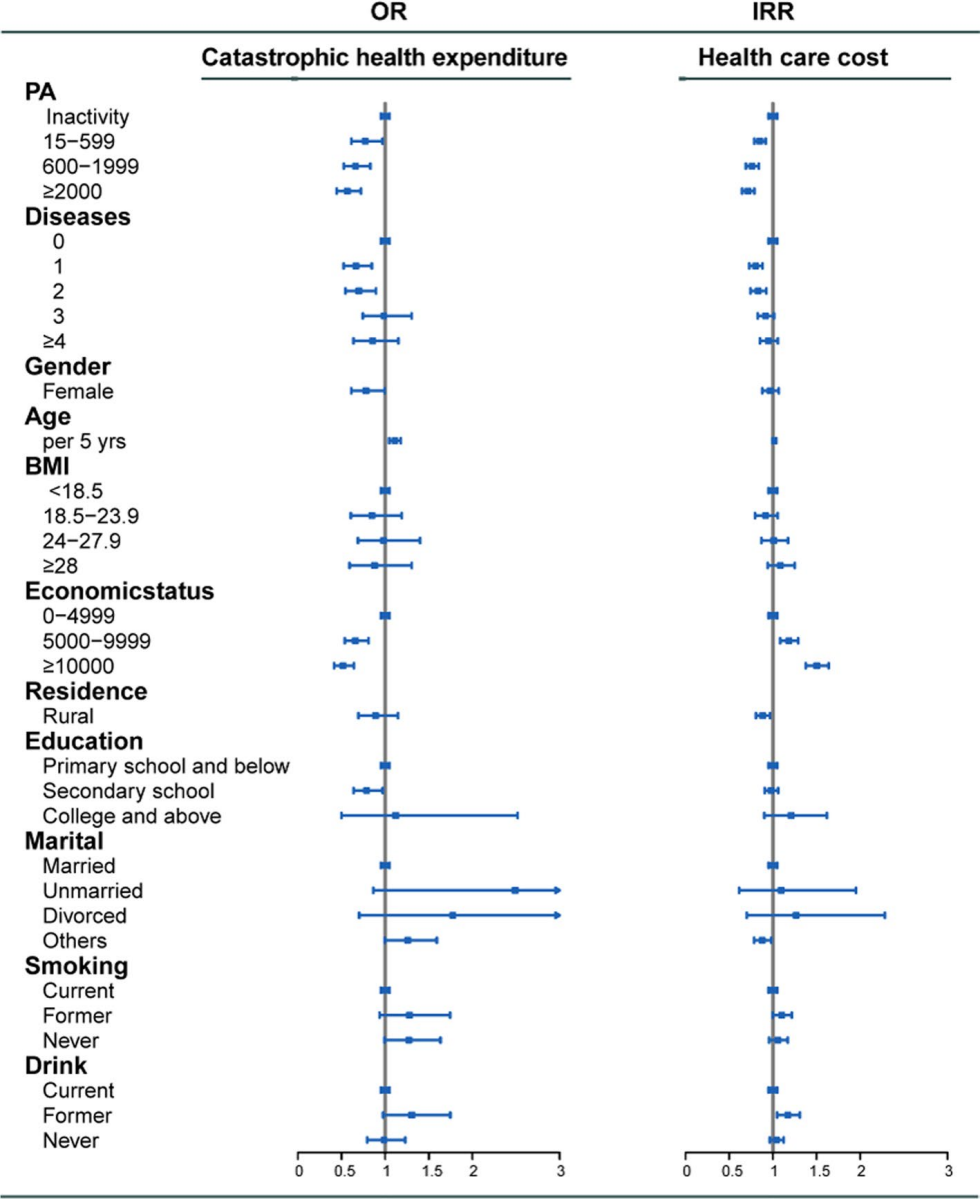
The association between physical activity and catastrophic health expenditure and health care cost (including outpatient service cost and cost of hospitalization), in China, 2011–2015. IRR was adjusted for age, sex, BMI, the number of non-communicable diseases, education, marital status, socioeconomic status, residence, smoking status, drinking status. Total time of moderate-intensity physical activity per week has been categorized into four levels: 0, 15–599, 600–1999, ≥ 2000 MET-minutes/week. IRR = incidence rate ratio

Since previous studies showed that TPA declined in China from 1991 to 2011 [5], we next used a Sankey diagram to visualize total time TPA for three time slices: 2011, 2013, and 2015. Random-effect logistic regression and negative binomial regression adjusted covariates described in the Methods were performed to further confirm the associations between different levels of TPA and healthcare utilization and expenditure, including CHE. Compared to inactive subjects, the highest level of TPA was significantly associated with decreased risk of inpatient visits (IRR: 0.58; 95% CI:0.50–0.67, *p* < 0.001), inpatient hospital days (IRR: 0.60; 95% CI: 0.42–0.84, *p* < 0.01), total healthcare cost (IRR: 0.71; 95% CI: 0.65–0.79, *p* < 0.001) and CHE (OR: 0.57; 95% CI: 0.45–0.72, *p* < 0.001) in Figs. 4 and 5.

Not only that, with the TPA level increasing, the risk for the cost of hospitalization and outpatient service cost were also in downward trend (S5 and S6 Figs) except for the number of outpatient visits (S4 Fig). These results indicated that engaging in higher levels of PA reduced the healthcare utilization and expenditure with a dose–response relationship.

## Discussion

This is the first study to try to unveil the connections of different amounts of PA with healthcare utilization and expenditure (including CHE) in China using a nationally representative data. We observed that persons engaged in TPA were usually accompanied with decreased healthcare utilization, total healthcare expenditure, and a lower risk of CHE. The higher level of TPA, the less healthcare utilization, total health expenditure and CHE. The present study provides novel evidence to support the health and economic value of PA. In terms of public health policy, it also provides a further rationale for prioritizing the promotion of physical activity as part of an integrated strategy to cut down the financial burden of chronic diseases.

Being physically active is one of the beneficial behaviors that persons of all ages can engage in to improve their health since the benefits of PA in reducing morbidity and mortality are well-established [1–4, 14, 15]. In addition, the health benefits of PA are extensive, such as improved sleep and quality of life, reduced anxiety, depression and fall risk, and improved cognitive function. In our study, we found that a beneficial association between TPA and the number of physical multimorbidity, defined as 12 non-communicable diseases, starts from a low dose, and performing highly recommended levels of PA with additional health benefits according to 2015 CHARLS.

Regardless of the health benefits of PA, insufficiently PA is a universal public health problem worldwide [2, 4–6, 16–18]. We found that more than one-third of Chinese over 50 years old never participate in any moderate or intense activity in 2015. Other studies also reported that 31.1% of adults are lack of exercise worldwide, with proportions ranging from 17.0% in southeast Asia to about 43% in the Americas and the eastern Mediterranean [18]. The pandemic of physical inactivity is known to create a huge healthcare and economic burden in terms of morbidity and mortality [6, 10, 17]. Therefore, engaging in PA is key to limit morbidity and death, and decrease the burden of aging populations worldwide.

In the current study, we first found that compared with inactive subjects, those who engaged in PA had a reduced total healthcare utilization, including inpatient visits and the average length of stay. Our findings are consistent with several recent reports, which showed the health benefits of PA [19–21]. Dohrn, et al. found that the higher the overall PA count accompanied with the lower risk of future inpatient and outpatient visits, and more sedentary time spent doubled the venture of hospital stays [19]. Alcaraz-Serrano V, et al. reported that individuals who spent more than 7.8 h per day in sedentariness gained an increased risk of hospitalization length resulting from bronchiectasis exacerbation [20]. More importantly, our novel findings suggest that implementing high levels of PA reduced the total health-care utilization of Chinese individuals with a dose–response relationship.

Several previous studies have looked at the financial burden of reduced physical activity at local, state or national levels in developed countries, but only two studies have shown its effect on the economic burden of China in 2006 and 2013, respectively [5, 7, 17]. Yang, et al. indicated that higher levels of PA were accompanied with lower healthcare costs among 483 Japanese elderly regardless of physical performance [22]. Using a countrywide typical sample of China adults for the 2011–2015 period, we found that compared to inactive individuals, the expenses of outpatient and inpatient in physical active subjects decreased significantly with the increase of PA. In addition, engaging in higher levels of PA reduced the total healthcare expenditure with a dose–response relationship in Chinese populations. One of the most interesting things is that PA can lead to a significantly reduced likelihood of CHE. The reasons for the decrease in medical costs are as follows: first, lower prevalence of comorbidity may contribute to the lower total health care expenses in persons with high physical activity; second, the reduced risk for outpatient visits and/or hospitalization in the exercise group reported in our study may explain their lower costs in health expenditure; in addition, people with high physical performance have a lower risk of geriatric syndromes such as sarcopenia, depression and fractures, and a higher life expectancy, all of which contribute to reducing the medical burden on society. Therefore, our study showed that appropriate promotion of the levels of PA can be a cost-effective method to ease the economic burden in China.

### Strengths and limitations

This present study has several important advantages. First, we used a large and countrywide typical cohort, despite of age, number of diseases, and socioeconomic status, which ensure the reliability and representativeness of the analysis conclusions. Second, this study is the first attempt to uncover the connections between TPA and healthcare utilization (including CHE) in China. Additionally, considering the dynamically changing of PA, we analyzed both the cross-sectional data from 2015 and three longitudinal follow-up data from 2011 to 2015, and all of which confirmed the benefits of TPA on health service use and healthcare expenditure.

There are, however, some limitations that need to be recognized. First, some unknown confounding factors might have affected the outcomes, regardless of statistical adjustments in the observational study. Second, the intensity, frequency, and duration of PA per week are self-reported, thus subjective measurements would result in biased estimates of the association between TPA and healthcare utilization. Finally, our study did not assess indirect costs, which are associated with productivity loss driven by disability and premature mortality due to illness.

Our results are based on comparing the healthcare utilization of people with or without physical activity, and the comparison groups are all with or without non-communicable diseases. As is well known, NCDs free people are likely to exercise more, while those who were disabled earlier by NCDs are less likely to exercise. Additionally, calculation method in this study may not provide an exact estimate of TPA in MET-minutes per week. It is worth noting that our study is also limited because the current research data is relatively old, and the current physical activity of Chinese people is affected by the government's COVID-19 policy.

## Conclusion

In conclusion, Engaging in moderate-to-vigorous PA may drive a potential decrease in healthcare utilization, healthcare expenditure and household financial risk with a dose–response relationship in China. Our findings indicate that advocating and promoting exercise in public health policy may be beneficial in reducing the burden of individuals and social healthcare systems.

### Supplementary Information

**Additional file 1:** **S1 Table.** Basic information about the participants. **Supplementary fig 1.** Restricted cubic splines to flexibly model adjusted covariates (age, sex, BMI, the number of non-communicable diseases, marital status, education, residence, socioeconomic status, smoking status, drinking status) showed  the association between the time of TPA per week and risk of the number of outpatient visits in China 2015. IRR=incidence rate ratio. **Supplementary fig 2.** Restricted cubic splines to flexibly model adjusted covariates (age, sex, BMI, the number of non-communicable diseases, marital status, education, residence, socioeconomic status, smoking status, drinking status) showed the association between the time of TPA per week and the risk of outpatient service cost in China 2015. IRR=incidence rate ratio. **Supplementary fig 3.** Restricted cubic splines to flexibly model adjusted covariates (age, sex, BMI, the number of non-communicable diseases, marital status, education, residence, socioeconomic status, smoking status, drinking status) showed the association between the time of TPA per week and risk of  the cost of hospitalization in China 2015. IRR=incidence rate ratio. **Supplementary fig 4.** Sankey diagram to visualize total time of moderate-intensity physical activity per week. 2300 objects had full  physical activity records for three time slices: 2011, 2013, and 2015. **Supplementary fig 5.** The association between physical activity and number of outpatient visits. **Supplementary fig 6.  **The association between physical activity and outpatient service cost. **Supplementary fig 7. **The association between physical activity and cost of hospitalization.

## Data Availability

The dataset supporting the conclusions of this article is available in the China Health and Retirement Longitudinal Study repository, http://charls.pku.edu.cn/index/zh-cn.html.
