# Supplementary material for: The role of physical activity on healthcare utilization in China
Source: BMC Public Health. 2023 Nov 30;23:2378. doi: 10.1186/s12889-023-16625-4 (PMC10691091; doi:10.1186/s12889-023-16625-4)
Supplement: Supplementary file 1 — Additional file 1: S1 Table. Basic information about the participants. Supplementary fig 1. Restricted cubic splines to flexibly model adjusted covariates (age, sex, BMI, the number of non-communicable diseases, marital status, education, residence, socioeconomic status, smoking status, drinking status) showed the association between the time of TPA per week and risk of the number of outpatient visits in China 2015. IRR=incidence rate ratio. Supplementary fig 2. Restricted cubic splines to flexibly model adjusted covariates (age, sex, BMI, the number of non-communicable diseases, marital status, education, residence, socioeconomic status, smoking status, drinking status) showed the association between the time of TPA per week and the risk of outpatient service cost in China 2015. IRR=incidence rate ratio. Supplementary fig 3. Restricted cubic splines to flexibly model adjusted covariates (age, sex, BMI, the number of non-communicable diseases, marital status, education, residence, socioeconomic status, smoking status, drinking status) showed the association between the time of TPA per week and risk of the cost of hospitalization in China 2015. IRR=incidence rate ratio. Supplementary fig 4. Sankey diagram to visualize total time of moderate-intensity physical activity per week. 2300 objects had full physical activity records for three time slices: 2011, 2013, and 2015. Supplementary fig 5. The association between physical activity and number of outpatient visits. Supplementary fig 6. The association between physical activity and outpatient service cost. Supplementary fig 7. The association between physical activity and cost of hospitalization. [file 12889_2023_16625_MOESM1_ESM.docx]

**S1 Table.** Basic information about the participants.

| **Variables** | **Total (n = 37770)** | **2011 (n = 12590)** | **2013 (n = 12590)** | **2015 (n = 12590)** |
| --- | --- | --- | --- | --- |
| **TPA, n (%)** |  |  |  |  |
| Inactivity | 5469 (36) | 1712 (34) | 1529 (37) | 2228 (38) |
| 15-599 | 2787 (18) | 818 (16) | 796 (19) | 1173 (20) |
| 600-1999 | 3293 (22) | 1140 (23) | 917 (22) | 1236 (21) |
| ≥2000 | 3581 (24) | 1354 (27) | 944 (23) | 1283 (22) |
| **TPA** | 525 (0, 1815) | 540 (0, 2400) | 525 (0, 1680) | 525 (0, 1680) |
| Median (IQR) |  |  |  |  |
| **Age, n (%)** |  |  |  |  |
| 45-49 | 3164 (8) | 2192 (17) | 972 (8) | 0 (0) |
| 50-54 | 6683 (18) | 1944 (15) | 2230 (18) | 2509 (20) |
| 55-59 | 7556 (20) | 2772 (22) | 2609 (21) | 2175 (17) |
| 60-64 | 7754 (21) | 2328 (18) | 2622 (21) | 2804 (22) |
| 65-69 | 5342 (14) | 1471 (12) | 1756 (14) | 2115 (17) |
| 70-74 | 3580 (9) | 1005 (8) | 1170 (9) | 1405 (11) |
| 75- | 3691 (10) | 878 (7) | 1231 (10) | 1582 (13) |
| **Age** |  |  |  |  |
| Median (IQR) | 60 (54, 67) | 58 (52, 65) | 60 (54, 67) | 62 (56, 69) |
| **Gender, n (%)** |  |  |  |  |
| Male | 18258 (48) | 6086 (48) | 6086 (48) | 6086 (48) |
| Female | 19488 (52) | 6496 (52) | 6496 (52) | 6496 (52) |
| **BMI, n (%)** |  |  |  |  |
| <18.5 | 1969 (7) | 697 (7) | 566 (6) | 706 (7) |
| 18.5-23.9 | 15317 (51) | 5476 (53) | 4725 (50) | 5116 (49) |
| 24-27.9 | 9239 (31) | 2957 (29) | 2992 (32) | 3290 (32) |
| ≥28 | 3652 (12) | 1177 (11) | 1190 (13) | 1285 (12) |
| **Number of diseases, n (%)** |  |  |  |  |
| 0 | 11675 (31) | 4149 (33) | 4199 (33) | 3327 (26) |
| 1 | 11107 (29) | 3776 (30) | 3748 (30) | 3583 (28) |
| 2 | 7516 (20) | 2404 (19) | 2389 (19) | 2723 (22) |
| 3 | 4086 (11) | 1280 (10) | 1277 (10) | 1529 (12) |
| 4 - | 3386 (9) | 981 (8) | 977 (8) | 1428 (11) |
| **Economicstatus, n (%)** |  |  |  |  |
| 0-4999 | 10794 (41) | 5875 (47) | 2389 (34) | 2530 (35) |
| 5000-9999 | 8238 (31) | 3842 (31) | 2307 (33) | 2089 (29) |
| ≥10000 | 7432 (28) | 2654 (21) | 2251 (32) | 2527 (35) |
| **Residence, n (%)** |  |  |  |  |
| Urban | 6380 (17) | 2373 (19) | 2512 (20) | 1495 (12) |
| Rural | 31347 (83) | 10210 (81) | 10075 (80) | 11062 (88) |
| **Education, n (%)** |  |  |  |  |
| Primary school and below | 25808 (68) | 8602 (68) | 8602 (68) | 8604 (68) |
| Secondary school | 11307 (30) | 3769 (30) | 3769 (30) | 3769 (30) |
| College and above | 624 (2) | 208 (2) | 208 (2) | 208 (2) |
| **Drinking, n (%)** |  |  |  |  |
| Current | 12579 (34) | 4193 (34) | 4193 (34) | 4193 (34) |
| Former | 3087 (8) | 1029 (8) | 1029 (8) | 1029 (8) |
| Never | 21813 (58) | 7271 (58) | 7271 (58) | 7271 (58) |
| **Smoking, n (%)** |  |  |  |  |
| Current | 12097 (32) | 4018 (32) | 4018 (32) | 4061 (32) |
| Former | 2970 (8) | 990 (8) | 990 (8) | 990 (8) |
| Never | 22518 (60) | 7490 (60) | 7490 (60) | 7538 (60) |
| **Number of outpatient visits** |  |  |  |  |
| Median (IQR) | 0 (0, 0) | 0 (0, 0) | 0 (0, 0) | 0 (0, 0) |
| **Number of inpatient visits** |  |  |  |  |
| Median (IQR) | 1 (1, 2) | 1 (1, 1) | 1 (1, 2) | 1 (1, 2) |
| **inpatient hospital days** |  |  |  |  |
| Median (IQR) | 9 (6, 15) | 8 (6, 15) | 9 (7, 15) | 8 (6, 15) |
| **Health care cost** |  |  |  |  |
| Median (IQR) | 3600 (1200, 10000) | 2400 (800, 6500) | 3600 (1080, 10125) | 4800 (1800, 12600) |
| **Outpatient service cost** |  |  |  |  |
| Median (IQR) | 2400 (840, 7200) | 1800 (600, 4800) | 2640 (900, 7200) | 3600 (1200, 11640) |
| **Cost of hospitalization** |  |  |  |  |
| Median (IQR) | 5000 (2000, 10000) | 3200 (1437.5, 7000) | 5000 (2000, 10000) | 5600 (2800, 12000) |
| **Catastrophic health expenditure** |  |  |  |  |
| 0 | 4031 (56) | 1827 (61) | 1159 (55) | 1045 (49) |
| 1 | 3202 (44) | 1170 (39) | 946 (45) | 1086 (51) |

**Suplementary figures**


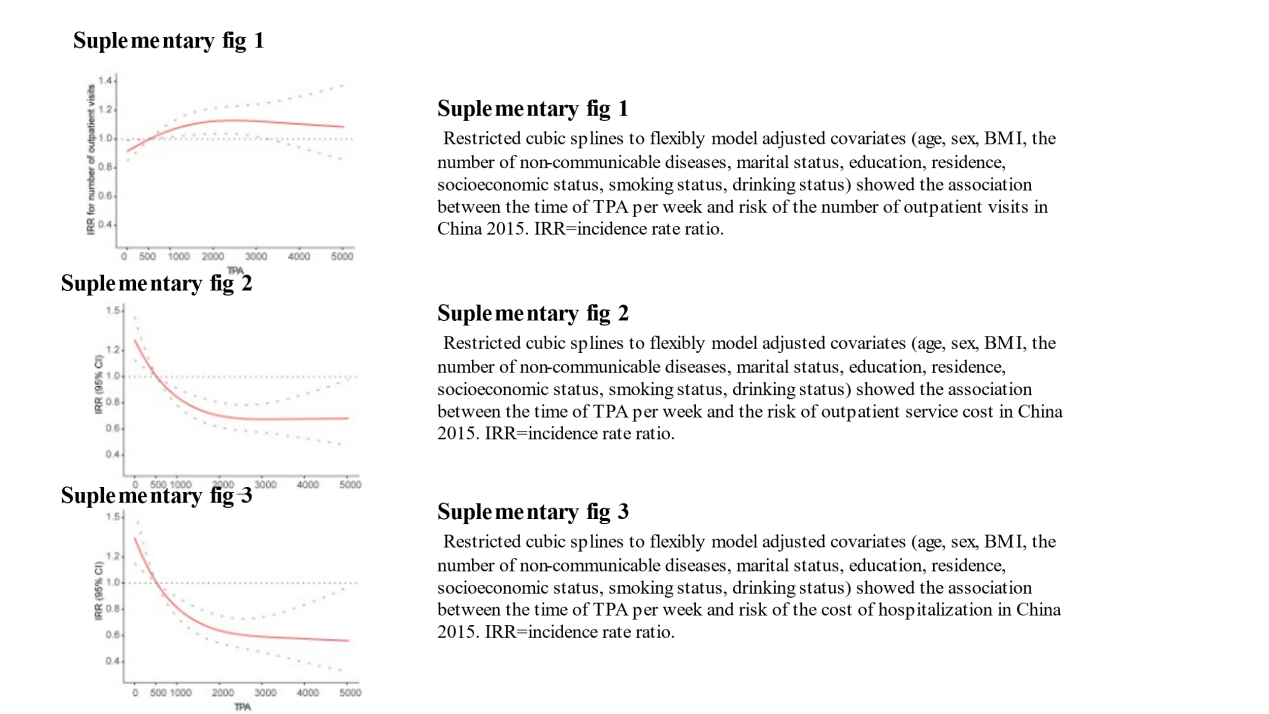


**Suplementary fig 1** Restricted cubic splines to flexibly model adjusted covariates (age, sex, BMI, the number of non-communicable diseases, marital status, education, residence, socioeconomic status, smoking status, drinking status) showed the association between the time of TPA per week and risk of the number of outpatient visits in China 2015. IRR=incidence rate ratio.


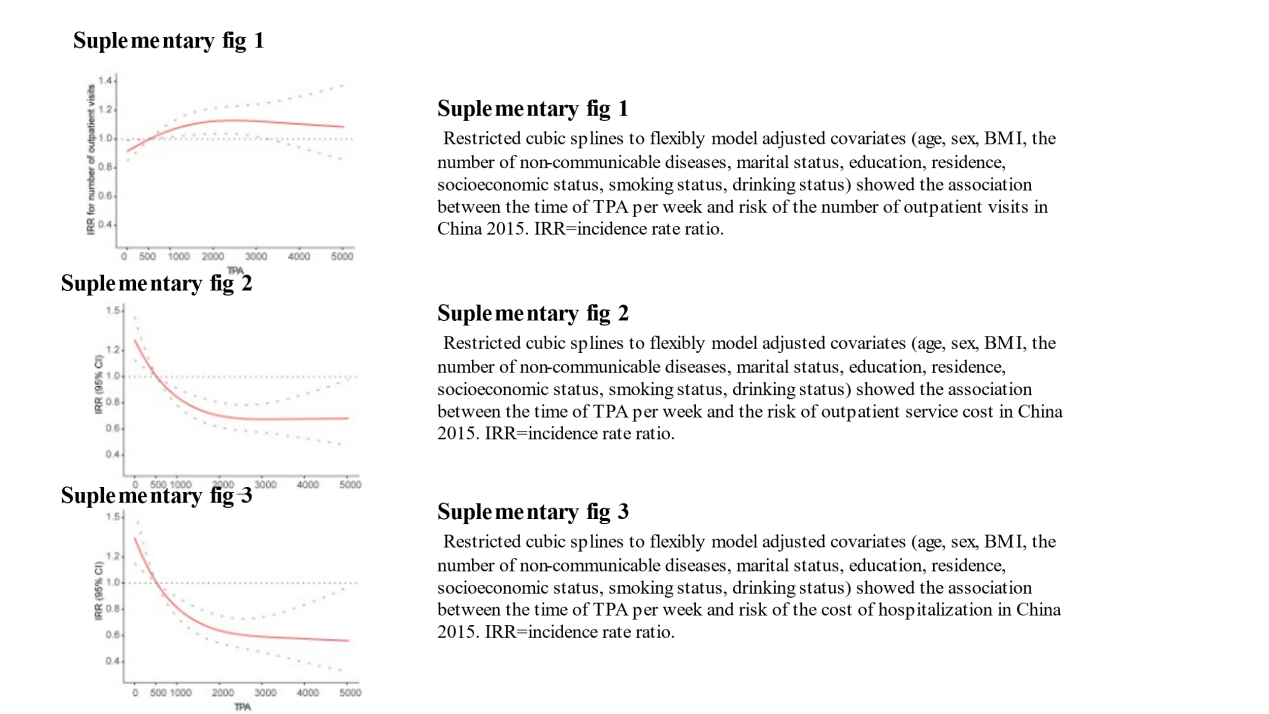


**Suplementary fig 2**

Restricted cubic splines to flexibly model adjusted covariates (age, sex, BMI, the number of non-communicable diseases, marital status, education, residence, socioeconomic status, smoking status, drinking status) showed the association between the time of TPA per week and the risk of outpatient service cost in China 2015. IRR=incidence rate ratio.


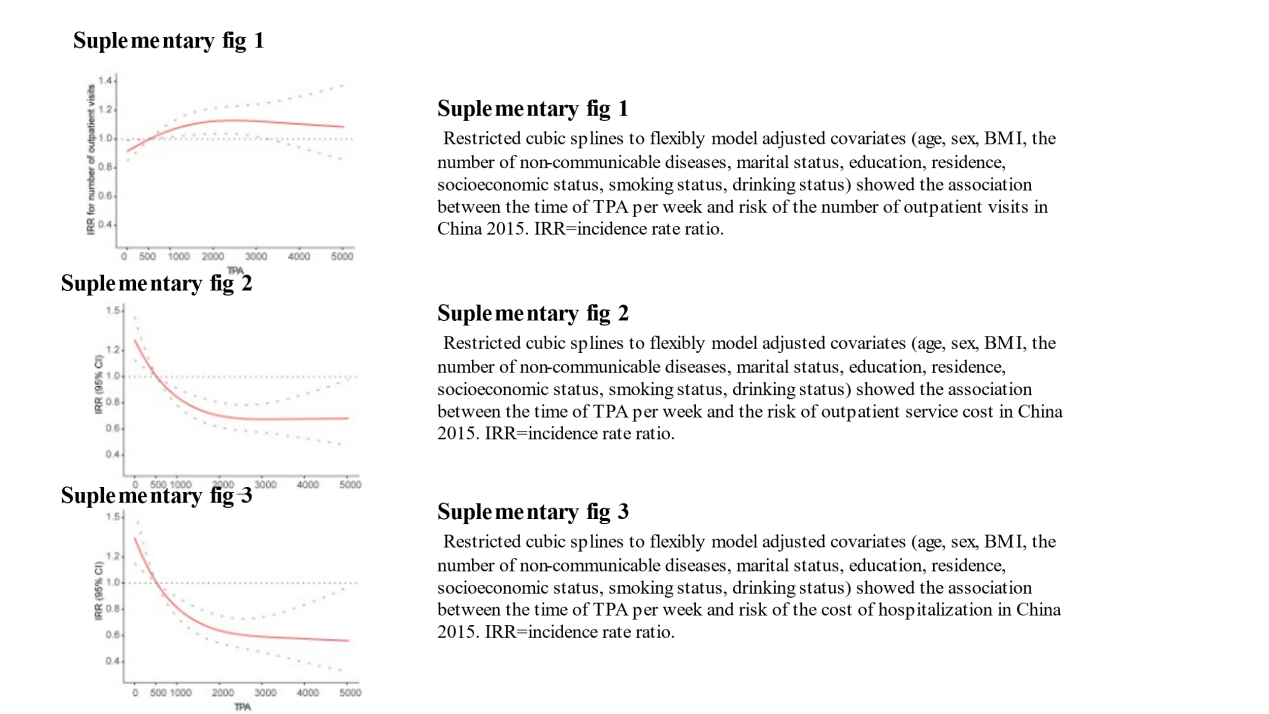


**Suplementary fig 3** Restricted cubic splines to flexibly model adjusted covariates (age, sex, BMI, the number of non-communicable diseases, marital status, education, residence, socioeconomic status, smoking status, drinking status) showed the association between the time of TPA per week and risk of the cost of hospitalization in China 2015. IRR=incidence rate ratio.


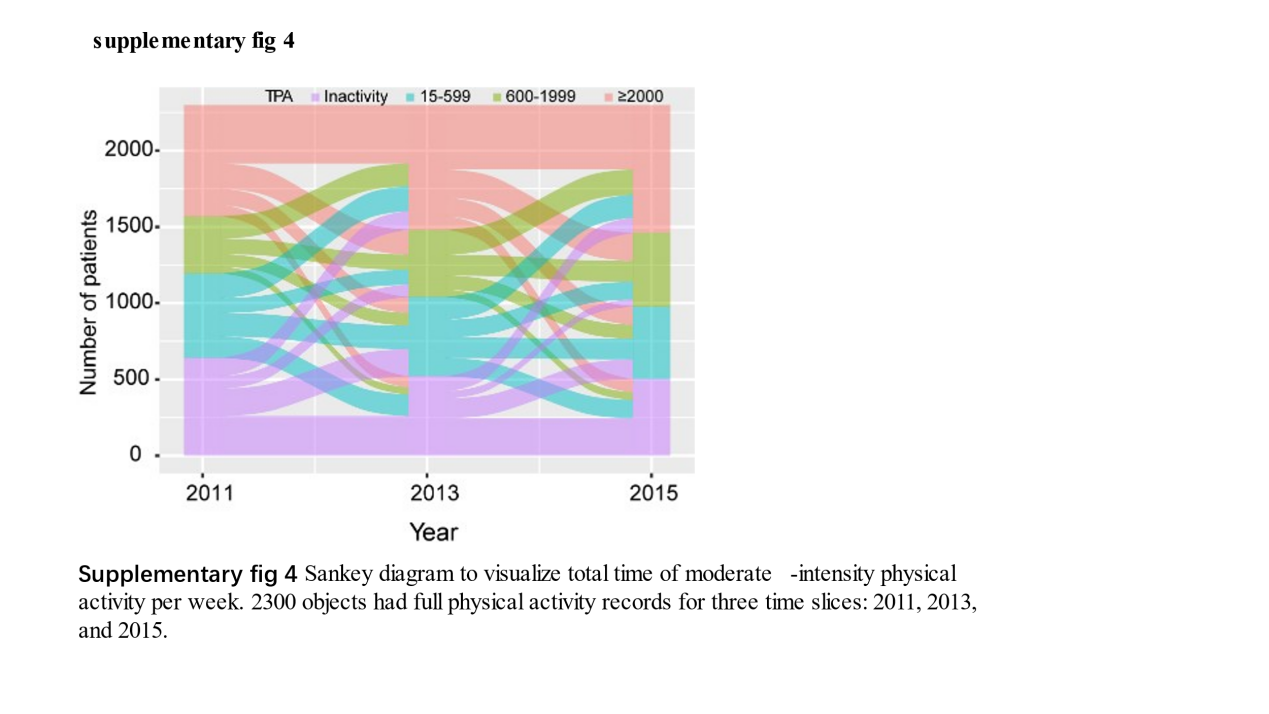


**Supplementary fig 4** Sankey diagram to visualize total time of moderate-intensity physical activity per week. 2300 objects had full physical activity records for three time slices: 2011, 2013, and 2015.


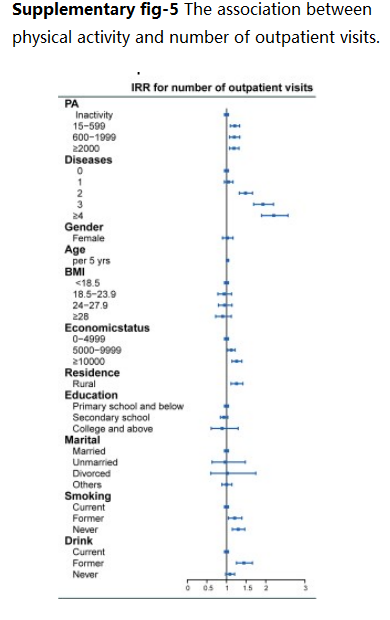


**Supplementary fig 5** The association between physical activity and number of outpatient visits.


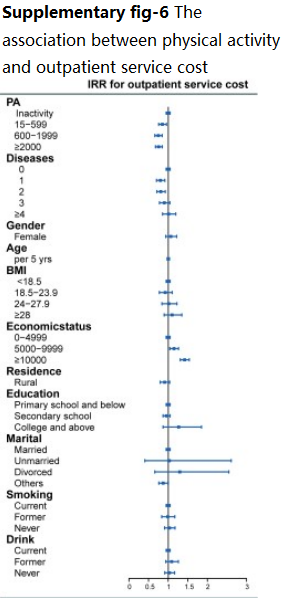


**Supplementary fig 6** The association between physical activity and outpatient service cost.


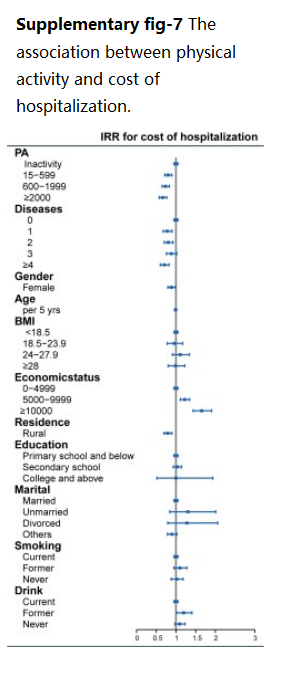


**Supplementary fig 7** The association between physical activity and cost of hospitalization
